# Performance Measurement for Surgery at National Level in China

**DOI:** 10.1002/hcs2.70066

**Published:** 2026-04-15

**Authors:** Tianyi Zhang, Qian Zhuang, Wei Liu, Yuehui Liu, Boya Zhang, Mingming Zhang, Xuan Zhang, Lin Li, Jianchao Liu, Zhouheng Ye

**Affiliations:** ^1^ Department of Medical Innovation and Research Institution of Hospital Management, Chinese PLA General Hospital Beijing China; ^2^ School of Humanities and Social Sciences North China Electric Power University Beijing China; ^3^ Department of Special Operations Medicine The Sixth Medical Center, Chinese PLA General Hospital Beijing China

**Keywords:** evaluation, performance measure, quality, surgery

## Abstract

**Background:**

Performance assessment is the first step to improve the quality of surgery. To date, no studies have systematically evaluated the surgical performance measures in China. This study aims to investigate the performance measures in surgical care at national level in China, with the objective of identifying existing gaps and potential opportunities in performance measurement of surgical care.

**Methods:**

We summarized and evaluated surgery related performance measures from five officially published performance evaluation indicator systems in China, and compared with those endorsed by National Quality Forum (NQF), the clearing house for all federal performance measures in the United States. The evaluation and comparison include: (1) the process of measure development; (2) the characteristics of the measures, including stewards, Donabedian frames, risk adjustment, and so on; (3) the keywords of the measures using word cloud analysis.

**Results:**

A total of 85 measures relevant to surgery were included in the analysis. Among them, the majority was outcome measures, which was similar to NQF measures (76.47% *vs.* 70.27%, *p* = 0.9875). The development of surgical performance measures in China was primarily led by the government, but lacked a third‐party evaluation mechanism. Further, compared with NQF, the performance measures were more generic and limited in specialty association engagement, risk adjustment, and payment linkage.

**Conclusions:**

While China has made significant progress in the performance measures in surgery, the opportunities for improvement remain in this area, including the involvement of special societies, the establishment of an evaluation mechanism, and the implementation of risk adjustment for measures.

## Graphical abstract

A total of 85 measures from 5 healthcare performance evaluation system in China were compared with 74 measures from the United States government‐recognized healthcare performance measures. In China, the keywords of performance measures for surgery mainly revolved around generic terms, such as “Surgical” and “Surgery.” While in the United States, the main keywords of outcome measures were complication, readmission, risk, and adjustment. There are areas for future improvement: (1) The involvement of special societies; (2) The establishment of an evaluation mechanism; and (3) The implementation of risk adjustment for measures.

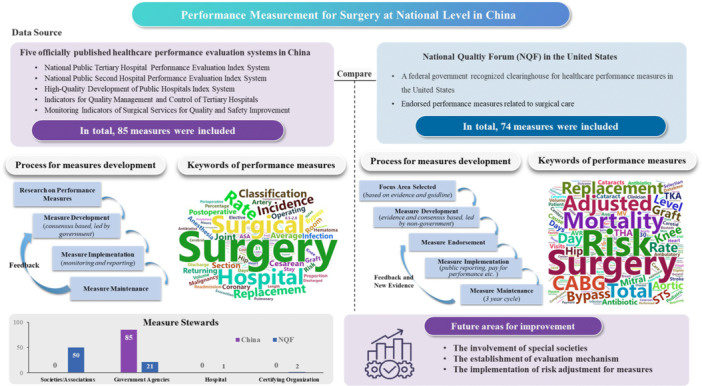

## 1 Introduction

Hospital performance assessment is increasingly important and has become a core discipline in the science of healthcare delivery because of its growing demand to ensure accountability and transparency, control healthcare costs, facilitate benchmarking, and improve the quality of healthcare [1, 2, 3]. In many healthcare systems, payment is progressively linked to performance measurements in pay‐for‐performance mechanisms, which means hospitals or doctors are reimbursed based on their performance in healthcare delivery [4, 5, 6, 7].

Performance of surgery has been a daunting prospect and the top medical conditions of interests of patients [8]. Given the increasing need and costs of surgery, performance measurement is more and more essential to surgical practice [9, 10]. In National Quality Forum (NQF), which is the federally designated performance measure endorsers (the work of measure endorsement was transferred to Battelle in March 2023 and renamed as Partnership for Quality Measurement) and its endorsed measures are considered the gold standard for health care measurement in the United States, the Surgery portfolio is one of the largest with more than 120 measures [9, 11, 12, 13].

In China, public hospitals play a dominant role in healthcare provision, delivering over 80% of medical services [14]. Since the healthcare reform in 2009, there has been a growing emphasis on performance assessments of public hospitals in China [15, 16, 17, 18, 19]. Various assessment systems have been introduced, including China Hospital Rankings by the Hospital Management Institute of Fudan University [20], China Hospital Competitiveness Ranking List by Hong Kong Alibi [21], the Science and technology evaluation metrics of hospitals by the Chinese academy of medical science [22], and China's Best Clinical Discipline Rankings by Peking University [19]. But these systems are mostly developed by third‐party organizations and mainly relied on reputation, science and technology output. In 2019, the Chinese government issued national public hospital performance evaluation index system for tertiary and secondary hospitals, respectively [23, 24], with a shift in focus towards daily medical services based on medical information system. Subsequently, an index system for evaluating the high‐quality development of public hospitals was announced in 2022 [25]. Among these performance measurement systems, surgical‐related measures constitute an important component. In addition, the National Health Commission (NHC) in China released the “Indicators for Quality Management and Control of Tertiary Hospitals” in 2011 based on the research about the Chinese Hospital Quality Indicator System (CHQIS), which consisted seven dimensions and one was directly associated with surgery [26, 27]. In 2023, the NHC specifically targeted surgical services and issued an action plan and monitoring indicators for quality and safety improvement [28].

Examining and analyzing the existing performance measures for surgery is the first step towards developing more robust and accurate methods for assessing the surgical care provided by hospitals. However, there have been no reported studies on surgery performance measurement in China by far. Therefore, this study is to comprehensively analyze the current performance measures for surgery developed by the Chinese government and compare them to NQF‐endorsed measures in the United States. This analysis and comparison include the process for developing performance measures, measures relevant to surgical care corresponding to the Donabedian quality framework (structure, process, and outcome) [29], and the keywords used in surgery performance measurements. Our study aims to identify gaps in performance measurements for surgery, inform current and future policies and practices for assessing surgical care, and support hospitals in providing value‐based, patient‐centered care.

## 2 Methods

### 2.1 Data Source

This study was designed to curate a comprehensive snapshot and landscape of current performance measures for surgery officially released by the Chinese government. Thus, a systematic review was conducted to identify performance measurements relevant to surgical care from (1) National Public Tertiary Hospital Performance Evaluation Index System (2023 version) [30]; (2) National Public Second Hospital Performance Evaluation Index System (2023 version) [31]; (3) High‐Quality Development of Public Hospitals Index System [25]; (4) Indicators for Quality Management and Control of Tertiary Hospitals [27]; and (5) Monitoring Indicators of Surgical Services for Quality and Safety Improvement [28]. As for the surgery performance measures in the United States, we refer to the NQF‐endorsed measures, which are a federal government‐recognized clearinghouse for performance measures [12].

### 2.2 Data Abstraction

The following information was abstracted into standardized forms for analysis: measure's name, steward, setting, data source, level of analysis, subspecialties associated, and reporting purpose (such as pay‐for‐performance, public reporting, and quality improvement). Further, we classified the measures according to the Donabedian conceptual model, which encompasses the domains of structure, process, and outcome. Structure measures reflect the attributes of the service or provider (e.g., participation in a systematic national database for general thoracic surgery, proportion of discharged patients undergoing IV level surgery); Process measures are defined as the components of care delivered, which typically align with generally accepted recommendations for clinical practice (e.g., percentage of antibacterial prophylaxis in clean surgical procedures, use of internal mammary artery in coronary artery bypass graft); Outcome measures indicate the health status of patients or end results of health care service (e.g., complication rate of patients undergoing surgery, risk‐adjusted operative mortality for aortic valve replacement). Data extraction and quality assessment were independently conducted by two investigators (T.Y.Z. and Q.Z.). Any disagreement between the two authors was resolved by discussion. If consensus could not be reached, the principal investigator (Z.H.Y.) made the final judgment.

### 2.3 Statistical Analysis

The characteristics of performance measures in surgical care were first described as a count (percentage). Subsequently, Chi‐square tests were employed to compare the difference between performance measures used in China and the United States. Lastly, co‐word analyses were conducted, and word clouds were constructed for measures in China and the United States, respectively, to identify the keywords of the performance measures based on the word frequencies [32].

A two‐tailed *p*‐value of less than 0.05 was considered statistically significant. Statistical analyses were executed using *R*, version 4.0.2 (R Project for Statistical Computing).

## 3 Results

### 3.1 Process for Performance Measures Development

**Figure 1:**
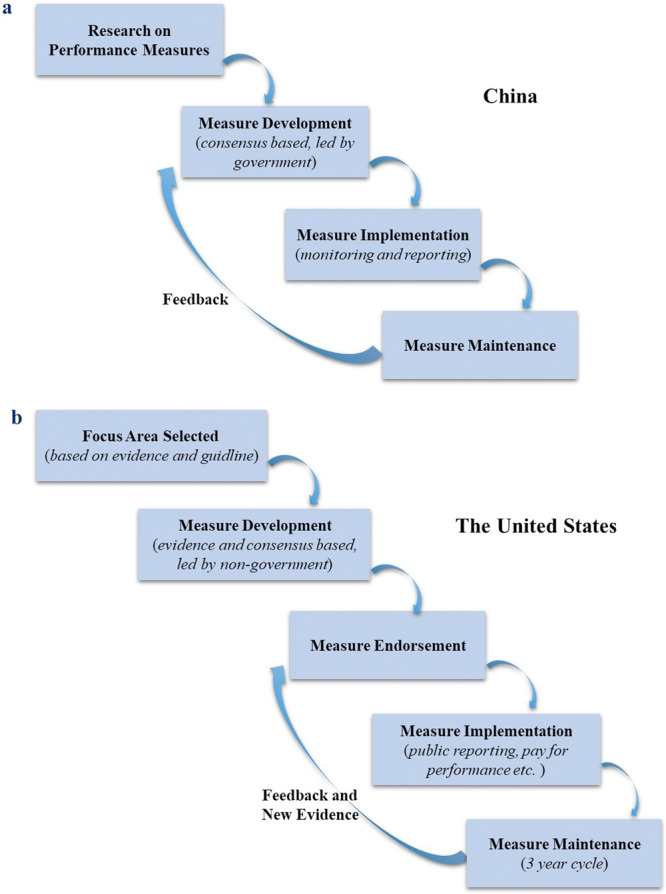
Process of performance measures development. (a) Process of performance measures development in China; (b) Process of performance measures development in the United States.

For National Public Tertiary and Secondary Hospital Performance Evaluation Index Systems, the performance measures were developed based on research led by the government and consensus among experts from official healthcare departments, academic and research institutes, and public hospitals. These measures are then monitored, analyzed, and reported annually. Further, measures were updated based on the feedback from various channels including on‐site investigations, conference discussions, official platform, and self‐assessment reports [30, 31]. On the basis above, the High‐Quality Development of Public Hospitals Index System was established according to three principles: refinement, feasibility, and measurability [25]. The Indicators for Quality Management and Control of Tertiary Hospitals were developed based on the research of CHQIS led by the NHC in China [26, 27]. However, the indicators have not been updated until the “Comprehensive Enhancement of Medical Quality Action Plan (2023–2025)” was proposed in 2023, which included an initiative specially to improve quality of surgical care and performance measures targeted to surgical care [28] (Figure 1a).

In the United States, the performance measures were initially developed by a measure steward (such as specialty society, hospital, healthcare quality improvement organization, and so on). A steward usually begins with identifying a focus area in which an improvement is needed after a thorough review of guideline and evidence. Then the steward establishes a work group consisting of experts in various fields to develop performance measures in focus area through an evidence‐based and consensus‐based process. After that, the steward submits the developed measures to NQF for endorsement. The NQF would convene a steering committee or a technical advisory panel to review and vote the measures according five standard criteria: (1) importance; (2) reliability and validity; (3) usability; (4) feasibility; and (5) competing and related measures. Once a measure developed or endorsed, the measure can be used in public reporting, pay for performance, or quality improvement. Further, endorsed measures undergo maintenance review every 3 years to determine whether they should be kept, modified, or retired [8, 12, 33] (Figure 1b).

### 3.2 Performance Measures for Surgical Care

The National Public Tertiary Hospital Performance Evaluation Index System comprises 55 measures across 4 dimensions: medical quality, operation efficiency, sustainable development, and patient satisfaction. Among these, measures on medical quality are placed greater emphasis, accounting for 43% of the total evaluation weight. Of the 55 measures, 7 related to surgical care, including proportion of discharged patients undergoing surgery, proportion of ambulatory (day) surgeries in all elective surgeries, proportion of discharged patients undergoing minimally invasive surgery, proportion of discharged patients undergoing IV level surgery, complication rate of patients undergoing surgery, infection rate of Type I surgical incision site, quality control for specific diagnosis or procedure. The measure of quality control for specific diagnosis or procedure can be further decomposed into 16 measurable surgery performance measures (since the National Public Tertiary Hospital Performance Evaluation Index System (2023 version) did not define the specific diagnosis and procedure, we referred to 2020 version). Thus, there were a total of 22 measures relevant to surgical care in this system. The National Public Secondary Hospital Performance Evaluation Index System included 28 measures across the same 4 dimensions. Among them, 4 measures were for surgical care. High‐Quality Development of Public Hospitals Index System borrowed five measures from the National Public Hospital Performance Evaluation Index System to evaluate the performance of surgical care. Additionally, 63 measures in Indicators for Quality Management and Control of Tertiary Hospitals and 8 in Monitoring Indicators of Surgical Services for Quality and Safety Improvement were related to surgery performance. In total, there were 85 measures included in analysis (Table S1).

In the United States, NQF has endorsed 1168 performance measures and 156 of which were related to surgical care. Of 156 measures, 85 have been removed, 64 continued endorsement and 10 were in endorsed with reserve status. Only measures in the status of endorsed or endorsed with reserve were included in this study (Table S2).

In China, the performance measures for surgery were all stewarded by government agencies, whereas in the United States, societies played a larger role (67.57%) in the stewardship. Compared with China, the surgical performance measures in the United States rely more on data from registries (60.81% *vs.* 0.00%, *p* < 0.0001) and are more frequently used in payment and public reporting programs (63.51% *vs.* 27.06%, *p* < 0.0001) (Table 1). In China, the surgical measures were more often used in performance assessment (27.06% vs 1.35%, *p* < 0.0001) and quality improvement program (76.47% *vs.* 50.00%, *p* < 0.0001) (Table 1). In the United States, 56.76% of surgical measures were adjusted or stratified by risk factors using statistical models, whereas in China, all the surgical performance measures were calculated without risk adjustment or stratification. In addition, the analysis of all surgical measures was conducted at the facility level. While, in the United States, there were 72.97% and 70.27% of measures were analyzed at facility and clinician levels, respectively. Although indicators in both systems primarily focused on inpatient settings with no significant difference in setting covered, the indicators in China are all confined to the inpatient setting, whereas the United States system includes a few indicators for outpatient and urgent care settings. Despite these differences, no significant differences were observed in the performance measures between the two countries in terms of the Donabedian conceptual classification and specialty coverage.

**Table 1:** Characteristics of performance measures for surgery in China and the United States.

Characteristics	China *n* (%)	The United States *n* (%)	*p*‐value
Donabedian conceptual model			
Structure	10 (11.76)	4 (5.41)	0.9875
Process	10 (11.76)	18 (24.32)	
Outcome	65 (76.47)	52 (70.27)	
Measure stewards			
Societies/associations	0 (0.00)	50 (67.57)	< 0.0001
Government agencies	85 (100.00)	21 (28.38)	
Hospital	0 (0.00)	1 (1.35)	
Certifying organization	0 (0.00)	2 (2.70)	
Setting			
Inpatient/outpatient	0 (0.00)	10 (13.51)	0.8237
Inpatient	85 (100.00)	57 (77.03)	
Outpatient	0 (0.00)	5 (6.76)	
Urgent	0 (0.00)	2 (2.70)	
Data source (nonexclusive)			
Claims	22 (25.88)	25 (33.78)	0.2761
Registry	0 (0.00)	45 (60.81)	< 0.0001
Level of analysis (nonexclusive)			
Facility	85 (100.00)	54 (72.97)	< 0.0001
Clinician	0 (0.00)	52 (70.27)	< 0.0001
Risk adjustment			
Statistical risk model	0 (0.00)	37 (50.00)	< 0.0001
Stratification by risk category/subgroup	0 (0.00)	5 (6.76)	
Other	0 (0.00)	1 (1.35)	
No risk adjustment or risk stratification	85 (100.00)	31 (41.89)	
Current use (nonexclusive)			
Payment	0 (0.00)	26 (35.14)	< 0.0001
Public reporting	23 (27.06)	47 (63.51)	< 0.0001
Performance assessment	23 (27.06)	1 (1.35)	< 0.0001
Quality improvement	65 (76.47)	37 (50.00)	0.0005
Specialty			
Generic	39 (45.88)	6 (8.11)	0.6841
General	0 (0.00)	3 (4.05)	
Cardiac	11 (12.94)	37 (50.00)	
Thoracic	0 (0.00)	2 (2.70)	
Vascular	0 (0.00)	5 (6.76)	
Orthopedic	12 (14.12)	12 (16.22)	
Neurosurgery	4 (4.71)	0 (0.00)	
Ophthalmology	0 (0.00)	4 (5.41)	
Genitourinary	0 (0.00)	2 (2.70)	
Cancer	8 (9.41)	1 (1.35)	
Obstetric	10 (11.76)	2 (2.70)	
Pediatric	1 (1.18)	0 (0.00)	

*Note:* Data are presented as frequency (percentage).

### 3.3 Keywords of Performance Measures Relevant to Surgical Care

**Figure 2:**
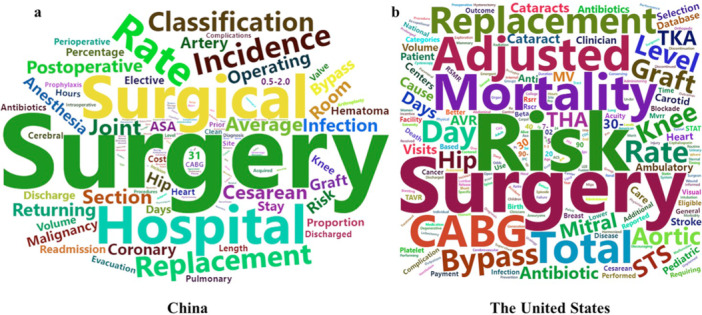
Key words of performance measures relevant to surgical care. (a) Key words of performance measures related surgery in China; (b) Keywords of performance measures related surgery the United States.

In China, the keywords of performance measures for surgical care mainly revolved around generic terms, such as “Surgical” and “Surgery”. The measures placed great emphasis on the outcome related to mortality, readmission, and return to the operating room. While in the United States, the measures were more specific, with a primary focus on cardiac and joint surgery, such as total knee arthroplasty (TKA) and coronary artery bypass grafting (CABG). The main keywords of outcome measures were complication and readmission. Additionally, “Risk” and “Adjustment” were also an important component of surgical measures, indicating the risk adjustment of performance measures received special attention in the United States (Figure 2).

## 4 Discussion

The assessment of surgical care performance is crucial and a top priority in supporting the delivery of high‐quality healthcare [34]. In our study, we conducted an analysis of 85 surgical performance measures developed by the Chinese government and compared them to the NQF library of performance measures, which is widely regarded as the gold standard for performance measurement in the United States. Our study indicates that China has placed increasing emphasis on the performance of surgical care, thereby encouraging hospitals, particularly tertiary hospitals, to provide more complex, minimally invasive, efficient, and higher quality surgical care. Furthermore, China has made significant advancements in the development of performance measures for surgery, including clearer definitions, data sources, and calculation methods. In addition, with the enhancement of information facilities in Chinese hospitals, the emphasis of performance evaluation has shifted towards the quality of healthcare provision, rather than academic reputation and research output.

In China, the government plays a pivotal role in the development of surgery performance measurement. Initially, the government leads collaborative research on healthcare performance assessment by coordinating administrative departments, universities, and hospitals. Subsequently, performance measures are established based on the research findings and consensus among management and clinical experts. This process and mechanism can promote communication and integration across various specialties and stakeholders [9], which makes the measures more generic and reduce the generation of redundant measures. However, the involvement of specialty societies and associations in this process was limited. The engagement of specialty in the development of performance measurement can render the measures more specific and better representative of the frontline of the surgical discipline [9]. Therefore, it is encouraged the participation of the specialty societies and establish a specialized agency, akin to NQF, to evaluate and endorse the performance measures developed by different organizations, while retaining the existing mechanism for generating performance measures.

The condition and severity of patient populations can influence the surgery outcomes in significant ways [35, 36]. So it is crucial to risk‐adjust the surgical measures, especially outcome measures, to ensure fair comparisons [37, 38, 39]. However, risk adjustment for surgical measures has not been widely implemented in China, and the underlying reasons have not been thoroughly explored. Several potential factors may contribute to this situation. First, the quality and standardization of data are still in need of improvement, which may affect the accuracy of risk adjustment and performance evaluation. Second, there is a lack of a nationally established, evidence‐based, validated, and localized risk adjustment model. Third, insufficient collaboration among clinical, data analysis, and IT departments restricts the development of a risk adjustment model for performance measurement.

Despite the rapid development of information systems in Chinese public hospitals in recent years, enabling performance measurement based on the daily healthcare delivery, the data sources mainly rely on claim data, which are not sufficiently comprehensive. In contrast, in the United States, 60.81% of surgical measures were calculated based on a registry database, which contains more extensive and comprehensive patient information, allowing for more accurate risk adjustment. Therefore, there is an urgent need for research on risk adjustment and the establishment of registration databases in China to enhance the comparability and equity of performance measurements for surgical care.

Although the link between performance and payment has not completely been established at national level in China, the performance assessment results have been used as important criteria for the resource allocation and appointment of leaders of public hospitals in some regional cities and provinces, such as Beijing, Shanghai, and Guangzhou [40, 41, 42]. Furthermore, the current surgical performance measures are mainly focused on the hospital level and not yet been extended to the clinician level. While in the United States, 70.27% of the surgical measures can be analyzed at the clinician level, with 30.77% of these measures linked to payment. However, the performance measurement at the surgeon level is controversial, since the strong dependence on accurate risk adjustment and limited sample size may lead to the risk of both gaming and cream‐skimming [43]. Thus, measuring performance at the surgeon level needs to be paid special caution and future research about it is required.

Another challenge of performance measurement in surgical care lies in meeting the demand for patient‐centered care, such as quality of life, shared decision‐making, and patient‐reported outcomes. For cancer patients undergoing surgical procedures, it is crucial to address long‐term patient‐centered outcomes, such as 5‐year survival, necessitating an extension of the follow‐up period for surgical patients. In addition, there is currently no consensus on the methods that can effectively combine various measures into a composite score to evaluate the overall performance of a hospital in surgical care [44]. The use of data‐driven analysis methods, such as machine‐learning algorithm [45], would be highly encouraged to provide novel composing, clustering, and visualization methods which are obscured in current surgical performance assessments.

The study has several limitations. First, this analysis only includes surgical performance measures that have been developed or led by government agencies, which may underrepresent institution/hospital‐developed metrics and hospital‐level quality monitoring. However, it enables a comprehensive reflection of surgical performance measurements at the national level, which is highly relevant to national policy‐making and healthcare system evaluations in China. Second, the measures we assessed would be subject to periodic revision with development of surgical technology. So the findings in our study provide a contemporary perspective that is historical inevitably. Finally, this study did not discuss the methodology of combining the various measures into composite scores due to the lack of consensus and transparency in the synthesis methods of the performance measures. Future research should address on studying the methodology of composing the various performance measures to assess the surgical performance comprehensively.

## 5 Conclusions

China has made remarkable progress in the development of performance measures of surgical care, with a shift in emphasis from academic reputation to the quality of healthcare delivery. Nevertheless, there remains ample room for improving the measurement of surgical performance, including risk adjustment, the engagement of medical specialty societies, the development of patient‐centered measures, and the methodology of comprehensively evaluating surgical performance by combining various individual performance measures.

## Author Contributions

**Tianyi Zhang:** conceptualization (equal), funding acquisition (equal), formal analysis (equal), project administration (equal), software (equal), validation (equal), visualization (equal), writing – original draft (equal). **Qian Zhuang:** formal analysis (equal), investigation (equal), project administration (equal), software (equal). **Jianchao Liu:** data curation (equal), formal analysis (equal), investigation (equal), project administration (equal), software (equal), validation (equal), writing – original draft (equal). **Wei Liu:** project administration (equal), validation (equal). **Yuehui Liu:** data curation (equal), project administration (equal). **Boya Zhang:** formal analysis (equal), project administration (equal). **Mingming Zhang:** project administration (equal). **Xuan Zhang:** investigation (equal), project administration (equal). **Lin Li:** conceptualization (equal), supervision (equal), validation (equal), writing – review and editing (equal). **Zhouheng Ye:** conceptualization (equal), formal analysis (equal), funding acquisition (equal), supervision (equal), validation (equal), writing – review and editing (equal).

## Ethics Statement

The authors have nothing to report.

## Consent

The authors have nothing to report.

## Conflicts of Interest

The authors declare no conflicts of interest.

## Supporting information


**Table S1:** Performance Measures in China.

**Table S2:** Performance Measures in the United States.

Table Caption for S1 and S2.

## Data Availability

The data of this paper are available upon request from the author.
