# Supplementary material for: Performance Measurement for Surgery at National Level in China
Source: Health Care Sci. 2026 Apr 15;5(2):109–16. doi: 10.1002/hcs2.70066 (PMC13109843; doi:10.1002/hcs2.70066)
Supplement: Supplementary file 1 — Table S1: Performance Measures in China. [file HCS2-5-109-s001.docx]

| **Table S1 Performance Measures for Surgery Issued by Official Agency in China** | | | | | | | | | |
| --- | --- | --- | --- | --- | --- | --- | --- | --- | --- |
| No | Measure | Data Source | Level of Analysis | Risk Adjustment | Setting of Care | Domains of Donabedian conceptual model | Current Use | Surgical Specialty | Steward |
| 1 | Proportion of discharged patients undergoing surgery | Cover page of Medical Record | Facility(Hospital) | No risk adjustment or risk stratification | Inpatient/Hospital | Structure | Performance assessment for secondary and tertiary hospital; Public reporting | Generic | National Public Tertiary Hospital Performance Evaluation Index System, National Public Second Hospital by National Health Commission |
| 2 | Proportion of ambulatory(day) surgeries in all elective surgeries | Reported and submitted by hospital itself | Facility(Hospital) | No risk adjustment or risk stratification | Inpatient/Hospital | Structure | Performance assessment for tertiary hospital; Public reporting; Quality surveillance and improvement | Generic | National Public Tertiary Hospital Performance Evaluation Index System Monitoring Indicators of Surgical Services for Quality and Safety Improvement by National Health Commission |
| 3 | Proportion of discharged patients undergoing minimally invasive surgery | Cover page of Medical Record | Facility(Hospital) | No risk adjustment or risk stratification | Inpatient/Hospital | Structure | Performance assessment for secondary and tertiary hospital; Public reporting | Generic | National Public Tertiary Hospital Performance Evaluation Index System National Public Second Hospital High-Quality Development of Public Hospitals Index System, by National Health Commission |
| 4 | Proportion of discharged patients undergoing IV level surgery | Cover page of Medical Record | Facility(Hospital) | No risk adjustment or risk stratification | Inpatient/Hospital | Structure | Performance assessment for tertiary hospital; Public reporting | Generic | National Public Tertiary Hospital Performance Evaluation Index System National Public Second Hospital High-Quality Development of Public Hospitals Index System, by National Health Commission |
| 5 | Proportion of discharged patients undergoing III level surgery | Cover page of Medical Record | Facility(Hospital) | No risk adjustment or risk stratification | Inpatient/Hospital | Structure | Performance assessment for secondary hospital; Public reporting | Generic | National Public Tertiary Hospital Performance Evaluation Index System National Public Second Hospital High-Quality Development of Public Hospitals Index System, by National Health Commission |
| 6 | Complication rate of patients undergoing surgery | Cover page of Medical Record | Facility(Hospital) | No risk adjustment or risk stratification | Inpatient/Hospital | Outcome | Performance assessment for secondary and tertiary hospital; Public reporting Quality surveillance and improvement | Generic | National Public Tertiary Hospital Performance Evaluation Index System National Public Second Hospital High-Quality Development of Public Hospitals Index System, Indicators for Quality Management and Control of Tertiary Hospitals Monitoring Indicators of Surgical Services for Quality and Safety Improvement by National Health Commission |
| 7 | Infection rate of Type I surgical incision (sterile incision) site | Cover page of Medical Record | Facility(Hospital) | No risk adjustment or risk stratification | Inpatient/Hospital | Outcome | Performance assessment for tertiary hospital; Public reporting | Generic | National Public Tertiary Hospital Performance Evaluation Index System National Public Second Hospital High-Quality Development of Public Hospitals Index System, by National Health Commission |
| 8 | Surgical volume of coronary artery bypass graft surgery | Cover page of Medical Record | Facility(Hospital) | No risk adjustment or risk stratification | Inpatient/Hospital | Structure | Performance assessment for tertiary hospital; Public reporting | Cardiac | National Public Tertiary Hospital Performance Evaluation Index System by National Health Commission |
| 9 | Surgical volume of hip joint replacement surgery | Cover page of Medical Record | Facility(Hospital) | No risk adjustment or risk stratification | Inpatient/Hospital | Structure | Performance assessment for tertiary hospital; Public reporting | Orthopedic | National Public Tertiary Hospital Performance Evaluation Index System by National Health Commission |
| 10 | Surgical volume of knee joint replacement surgery | Cover page of Medical Record | Facility(Hospital) | No risk adjustment or risk stratification | Inpatient/Hospital | Structure | Performance assessment for tertiary hospital; Public reporting | Orthopedic | National Public Tertiary Hospital Performance Evaluation Index System by National Health Commission |
| 11 | Surgical volume of cesarean section | Cover page of Medical Record | Facility(Hospital) | No risk adjustment or risk stratification | Inpatient/Hospital | Structure | Performance assessment for tertiary hospital; Public reporting | Obstetric | National Public Tertiary Hospital Performance Evaluation Index System by National Health Commission |
| 12 | Average length of stay of coronary artery bypass graft surgery | Cover page of Medical Record | Facility(Hospital) | No risk adjustment or risk stratification | Inpatient/Hospital | Outcome | Performance assessment for tertiary hospital; Public reporting | Cardiac | National Public Tertiary Hospital Performance Evaluation Index System by National Health Commission |
| 13 | Average length of stay of hip joint replacement surgery | Cover page of Medical Record | Facility(Hospital) | No risk adjustment or risk stratification | Inpatient/Hospital | Outcome | Performance assessment for tertiary hospital; Public reporting | Orthopedic | National Public Tertiary Hospital Performance Evaluation Index System by National Health Commission |
| 14 | Average length of stay of knee joint replacement surgery | Cover page of Medical Record | Facility(Hospital) | No risk adjustment or risk stratification | Inpatient/Hospital | Outcome | Performance assessment for tertiary hospital; Public reporting | Orthopedic | National Public Tertiary Hospital Performance Evaluation Index System by National Health Commission |
| 15 | Average length of stay of cesarean section | Cover page of Medical Record | Facility(Hospital) | No risk adjustment or risk stratification | Inpatient/Hospital | Outcome | Performance assessment for tertiary hospital; Public reporting | Obstetric | National Public Tertiary Hospital Performance Evaluation Index System by National Health Commission |
| 16 | Average cost of coronary artery bypass graft surgery | Cover page of Medical Record | Facility(Hospital) | No risk adjustment or risk stratification | Inpatient/Hospital | Outcome | Performance assessment for tertiary hospital; Public reporting | Cardiac | National Public Tertiary Hospital Performance Evaluation Index System by National Health Commission |
| 17 | Average cost of hip joint replacement surgery | Cover page of Medical Record | Facility(Hospital) | No risk adjustment or risk stratification | Inpatient/Hospital | Outcome | Performance assessment for tertiary hospital; Public reporting | Orthopedic | National Public Tertiary Hospital Performance Evaluation Index System by National Health Commission |
| 18 | Average cost of Stay of knee joint replacement surgery | Cover page of Medical Record | Facility(Hospital) | No risk adjustment or risk stratification | Inpatient/Hospital | Outcome | Performance assessment for tertiary hospital; Public reporting | Orthopedic | National Public Tertiary Hospital Performance Evaluation Index System by National Health Commission |
| 19 | Average cost of Stay of cesarean section | Cover page of Medical Record | Facility(Hospital) | No risk adjustment or risk stratification | Inpatient/Hospital | Outcome | Performance assessment for tertiary hospital; Public reporting | Obstetric | National Public Tertiary Hospital Performance Evaluation Index System by National Health Commission |
| 20 | In-hospital Mortality of Patients Undergoing coronary artery bypass graft surgery | Cover page of Medical Record | Facility(Hospital) | No risk adjustment or risk stratification | Inpatient/Hospital | Outcome | Performance assessment for tertiary hospital; Public reporting; Quality surveillance and improvement | Cardiac | National Public Tertiary Hospital Performance Evaluation Index System, Indicators for Quality Management and Control of Tertiary Hospitals by National Health Commission |
| 21 | In-hospital Mortality of Patients Undergoing hip joint replacement surgery | Cover page of Medical Record | Facility(Hospital) | No risk adjustment or risk stratification | Inpatient/Hospital | Outcome | Performance assessment for tertiary hospital; Public reporting | Orthopedic | National Public Tertiary Hospital Performance Evaluation Index System, Indicators for Quality Management and Control of Tertiary Hospitals by National Health Commission |
| 22 | In-hospital Mortality of Patients Undergoing knee joint replacement surgery | Cover page of Medical Record | Facility(Hospital) | No risk adjustment or risk stratification | Inpatient/Hospital | Outcome | Performance assessment for tertiary hospital; Public reporting | Orthopedic | National Public Tertiary Hospital Performance Evaluation Index System by National Health Commission |
| 23 | In-hospital Mortality of Patients Undergoing cesarean section | Cover page of Medical Record | Facility(Hospital) | No risk adjustment or risk stratification | Inpatient/Hospital | Outcome | Performance assessment for tertiary hospital; Public reporting | Obstetric | National Public Tertiary Hospital Performance Evaluation Index System, Indicators for Quality Management and Control of Tertiary Hospitals by National Health Commission |
| 24 | In-hospital Mortality in Neonatal Patients Undergoing Surgery | Not defined in public document | Facility(Hospital) | No risk adjustment or risk stratification | Inpatient/Hospital | Outcome | Quality surveillance and improvement | Pediatric | Indicators for Quality Management and Control of Tertiary Hospitals by National Health Commission |
| 25 | In-hospital mortality of surgical patients | Not defined in public document | Facility(Hospital) | No risk adjustment or risk stratification | Inpatient/Hospital | Outcome | Quality surveillance and improvement | Generic | Indicators for Quality Management and Control of Tertiary Hospitals by National Health Commission |
| 26 | Perioperative Mortality of Surgical Patients | Not defined in public document | Facility(Hospital) | No risk adjustment or risk stratification | Inpatient/Hospital | Outcome | Quality surveillance and improvement | Generic | Indicators for Quality Management and Control of Tertiary Hospitals Monitoring Indicators of Surgical Services for Quality and Safety Improvement by National Health Commission |
| 27 | Perioperative Mortality of Elective Surgery Patients | Not defined in public document | Facility(Hospital) | No risk adjustment or risk stratification | Inpatient/Hospital | Outcome | Quality surveillance and improvement | Generic | Indicators for Quality Management and Control of Tertiary Hospitals by National Health Commission |
| 28 | Perioperative Mortality by Anesthesia Risk Classification (ASA Classification) for Perioperative Patients | Not defined in public document | Facility(Hospital) | No risk adjustment or risk stratification | Inpatient/Hospital | Outcome | Quality surveillance and improvement | Generic | Indicators for Quality Management and Control of Tertiary Hospitals by National Health Commission |
| 29 | In-hospital Mortality of Patients with Surgical Complications | Not defined in public document | Facility(Hospital) | No risk adjustment or risk stratification | Inpatient/Hospital | Outcome | Quality surveillance and improvement | Generic | Indicators for Quality Management and Control of Tertiary Hospitals by National Health Commission |
| 30 | In-hospital Mortality of Patients Undergoing Cerebral Hematoma Evacuation Surgery | Not defined in public document | Facility(Hospital) | No risk adjustment or risk stratification | Inpatient/Hospital | Outcome | Quality surveillance and improvement | Neurosurgery | Indicators for Quality Management and Control of Tertiary Hospitals by National Health Commission |
| 31 | In-hospital Mortality of Cesarean Section Patients | Not defined in public document | Facility(Hospital) | No risk adjustment or risk stratification | Inpatient/Hospital | Outcome | Quality surveillance and improvement | Obstetric | Indicators for Quality Management and Control of Tertiary Hospitals by National Health Commission |
| 32 | In-hospital Mortality of Patients Undergoing Heart Valve Replacement Surgery | Not defined in public document | Facility(Hospital) | No risk adjustment or risk stratification | Inpatient/Hospital | Outcome | Quality surveillance and improvement | Cardiac | Indicators for Quality Management and Control of Tertiary Hospitals by National Health Commission |
| 33 | In-hospital Mortality of Patients Undergoing Surgery for Renal Malignancy | Not defined in public document | Facility(Hospital) | No risk adjustment or risk stratification | Inpatient/Hospital | Outcome | Quality surveillance and improvement | Cancer | Indicators for Quality Management and Control of Tertiary Hospitals by National Health Commission |
| 34 | In-hospital Mortality of Patients Undergoing Surgery for Hepatic Malignancy | Not defined in public document | Facility(Hospital) | No risk adjustment or risk stratification | Inpatient/Hospital | Outcome | Quality surveillance and improvement | Cancer | Indicators for Quality Management and Control of Tertiary Hospitals by National Health Commission |
| 35 | In-hospital Mortality of Patients Undergoing Surgery for Pulmonary Malignancy | Not defined in public document | Facility(Hospital) | No risk adjustment or risk stratification | Inpatient/Hospital | Outcome | Quality surveillance and improvement | Cancer | Indicators for Quality Management and Control of Tertiary Hospitals by National Health Commission |
| 36 | In-hospital Mortality of Patients Undergoing Surgery for Gastric Malignancy | Not defined in public document | Facility(Hospital) | No risk adjustment or risk stratification | Inpatient/Hospital | Outcome | Quality surveillance and improvement | Cancer | Indicators for Quality Management and Control of Tertiary Hospitals by National Health Commission |
| 37 | In-hospital Mortality of Patients Undergoing Surgery for Rectal Malignancy | Not defined in public document | Facility(Hospital) | No risk adjustment or risk stratification | Inpatient/Hospital | Outcome | Quality surveillance and improvement | Cancer | Indicators for Quality Management and Control of Tertiary Hospitals by National Health Commission |
| 38 | In-hospital Mortality of Patients Undergoing Surgery for Colonic Malignancy | Not defined in public document | Facility(Hospital) | No risk adjustment or risk stratification | Inpatient/Hospital | Outcome | Quality surveillance and improvement | Cancer | Indicators for Quality Management and Control of Tertiary Hospitals by National Health Commission |
| 39 | In-hospital Mortality of Patients Returning to Operating Room | Not defined in public document | Facility(Hospital) | No risk adjustment or risk stratification | Inpatient/Hospital | Outcome | Quality surveillance and improvement | Generic | Indicators for Quality Management and Control of Tertiary Hospitals by National Health Commission |
| 40 | In-hospital Mortality by Anesthesia Risk Classification (ASA Classification) for Patients Undergoing Coronary Artery Bypass Graft (CABG) Surgery | Not defined in public document | Facility(Hospital) | No risk adjustment or risk stratification | Inpatient/Hospital | Outcome | Quality surveillance and improvement | Cardiac | Indicators for Quality Management and Control of Tertiary Hospitals by National Health Commission |
| 41 | In-hospital Mortality by Anesthesia Risk Classification (ASA Classification) for Patients Undergoing Cerebral Hematoma Evacuation Surgery | Not defined in public document | Facility(Hospital) | No risk adjustment or risk stratification | Inpatient/Hospital | Outcome | Quality surveillance and improvement | Neurosurgery | Indicators for Quality Management and Control of Tertiary Hospitals by National Health Commission |
| 42 | In-hospital Mortality by Anesthesia Risk Classification (ASA Classification) for Cesarean Section Patients | Not defined in public document | Facility(Hospital) | No risk adjustment or risk stratification | Inpatient/Hospital | Outcome | Quality surveillance and improvement | Obstetric | Indicators for Quality Management and Control of Tertiary Hospitals by National Health Commission |
| 43 | In-hospital Mortality by Anesthesia Risk Classification (ASA Classification) for Hip Arthroplasty Patients | Not defined in public document | Facility(Hospital) | No risk adjustment or risk stratification | Inpatient/Hospital | Outcome | Quality surveillance and improvement | Orthopedic | Indicators for Quality Management and Control of Tertiary Hospitals by National Health Commission |
| 44 | In-hospital Mortality by Anesthesia Risk Classification (ASA Classification) for Patients Undergoing Heart Valve Replacement Surgery | Not defined in public document | Facility(Hospital) | No risk adjustment or risk stratification | Inpatient/Hospital | Outcome | Quality surveillance and improvement | Cardiac | Indicators for Quality Management and Control of Tertiary Hospitals by National Health Commission |
| 45 | Readmission Rate within 31 Days after Discharge of Patients Undergoing Coronary Artery Bypass Graft (CABG) Surgery | Not defined in public document | Facility(Hospital) | No risk adjustment or risk stratification | Inpatient/Hospital | Outcome | Quality surveillance and improvement | Cardiac | Indicators for Quality Management and Control of Tertiary Hospitals by National Health Commission |
| 46 | Readmission Rate within 31 Days after Discharge of Hysterectomy Patients | Not defined in public document | Facility(Hospital) | No risk adjustment or risk stratification | Inpatient/Hospital | Outcome | Quality surveillance and improvement | Obstetric | Indicators for Quality Management and Control of Tertiary Hospitals by National Health Commission |
| 47 | Readmission Rate within 31 Days after Discharge of Cesarean Section Patients | Not defined in public document | Facility(Hospital) | No risk adjustment or risk stratification | Inpatient/Hospital | Outcome | Quality surveillance and improvement | Obstetric | Indicators for Quality Management and Control of Tertiary Hospitals by National Health Commission |
| 48 | Readmission Rate within 31 Days after Discharge of Heart Valve Replacement Surgery Patients | Not defined in public document | Facility(Hospital) | No risk adjustment or risk stratification | Inpatient/Hospital | Outcome | Quality surveillance and improvement | Cardiac | Indicators for Quality Management and Control of Tertiary Hospitals by National Health Commission |
| 49 | Readmission Rate within 31 Days after Discharge of Patients Undergoing Cerebral Hematoma Evacuation Surgery | Not defined in public document | Facility(Hospital) | No risk adjustment or risk stratification | Inpatient/Hospital | Outcome | Quality surveillance and improvement | Neurosurgery | Indicators for Quality Management and Control of Tertiary Hospitals by National Health Commission |
| 50 | Overall Rate of Patients Returning to Operating Room | Not defined in public document | Facility(Hospital) | No risk adjustment or risk stratification | Inpatient/Hospital | Outcome | Quality surveillance and improvement | Generic | Indicators for Quality Management and Control of Tertiary Hospitals,Monitoring Indicators of Surgical Services for Quality and Safety Improvement by National Health Commission |
| 51 | Rate of Coronary Artery Bypass Graft (CABG) Surgery Patients Returning to Operating Room | Not defined in public document | Facility(Hospital) | No risk adjustment or risk stratification | Inpatient/Hospital | Outcome | Quality surveillance and improvement | Cardiac | Indicators for Quality Management and Control of Tertiary Hospitals by National Health Commission |
| 52 | Rate of Patients Undergoing Cerebral Hematoma Evacuation Surgery Returning Operating Room | Not defined in public document | Facility(Hospital) | No risk adjustment or risk stratification | Inpatient/Hospital | Outcome | Quality surveillance and improvement | Neurosurgery | Indicators for Quality Management and Control of Tertiary Hospitals by National Health Commission |
| 53 | Rate of Cesarean Section Patients Returning to Operating Room | Not defined in public document | Facility(Hospital) | No risk adjustment or risk stratification | Inpatient/Hospital | Outcome | Quality surveillance and improvement | Obstetric | Indicators for Quality Management and Control of Tertiary Hospitals by National Health Commission |
| 54 | Rate of Hip Arthroplasty Patients Returning to Operating Room | Not defined in public document | Facility(Hospital) | No risk adjustment or risk stratification | Inpatient/Hospital | Outcome | Quality surveillance and improvement | Orthopedic | Indicators for Quality Management and Control of Tertiary Hospitals by National Health Commission |
| 55 | Rate of Patients Undergoing Heart Valve Replacement Surgery Returning to Operating Room | Not defined in public document | Facility(Hospital) | No risk adjustment or risk stratification | Inpatient/Hospital | Outcome | Quality surveillance and improvement | Cardiac | Indicators for Quality Management and Control of Tertiary Hospitals by National Health Commission |
| 56 | Rate of Elective Surgery Patients Returning to Operating Room | Not defined in public document | Facility(Hospital) | No risk adjustment or risk stratification | Inpatient/Hospital | Outcome | Quality surveillance and improvement | Generic | Indicators for Quality Management and Control of Tertiary Hospitals by National Health Commission |
| 57 | Surgery Associated Hospital-Acquired Infection Incidence | Not defined in public document | Facility(Hospital) | No risk adjustment or risk stratification | Inpatient/Hospital | Outcome | Quality surveillance and improvement | Generic | Indicators for Quality Management and Control of Tertiary Hospitals by National Health Commission |
| 58 | Pulmonary Infection in Surgical Patients | Not defined in public document | Facility(Hospital) | No risk adjustment or risk stratification | Inpatient/Hospital | Outcome | Quality surveillance and improvement | Generic | Indicators for Quality Management and Control of Tertiary Hospitals by National Health Commission |
| 59 | Surgical Site Infection | Not defined in public document | Facility(Hospital) | No risk adjustment or risk stratification | Inpatient/Hospital | Outcome | Quality surveillance and improvement | Generic | Indicators for Quality Management and Control of Tertiary Hospitals by National Health Commission |
| 60 | Hospital-Acquired Infection Incidence in Elective Surgery Patients | Not defined in public document | Facility(Hospital) | No risk adjustment or risk stratification | Inpatient/Hospital | Outcome | Quality surveillance and improvement | Generic | Indicators for Quality Management and Control of Tertiary Hospitals by National Health Commission |
| 61 | Pulmonary Infection Incidence in Elective Surgery Patients | Not defined in public document | Facility(Hospital) | No risk adjustment or risk stratification | Inpatient/Hospital | Outcome | Quality surveillance and improvement | Generic | Indicators for Quality Management and Control of Tertiary Hospitals by National Health Commission |
| 62 | Surgical Site Infection Classified by National Nosocomial Infection Surveillance (NNIS) Risk Index | Not defined in public document | Facility(Hospital) | No risk adjustment or risk stratification | Inpatient/Hospital | Outcome | Quality surveillance and improvement | Generic | Indicators for Quality Management and Control of Tertiary Hospitals by National Health Commission |
| 63 | Postoperative Pulmonary Embolism Incidence in Surgical Patients | Not defined in public document | Facility(Hospital) | No risk adjustment or risk stratification | Inpatient/Hospital | Outcome | Quality surveillance and improvement | Generic | Indicators for Quality Management and Control of Tertiary Hospitals by National Health Commission |
| 64 | Postoperative Deep Vein Thromboembolism Incidence in Surgical Patients | Not defined in public document | Facility(Hospital) | No risk adjustment or risk stratification | Inpatient/Hospital | Outcome | Quality surveillance and improvement | Generic | Indicators for Quality Management and Control of Tertiary Hospitals,Monitoring Indicators of Surgical Services for Quality and Safety Improvement by National Health Commission |
| 65 | Postoperative Sepsis Incidence in Surgical Patients | Not defined in public document | Facility(Hospital) | No risk adjustment or risk stratification | Inpatient/Hospital | Outcome | Quality surveillance and improvement | Generic | Indicators for Quality Management and Control of Tertiary Hospitals by National Health Commission |
| 66 | Postoperative Hemorrhage or Hematoma Incidence in Surgical Patients | Not defined in public document | Facility(Hospital) | No risk adjustment or risk stratification | Inpatient/Hospital | Outcome | Quality surveillance and improvement | Generic | Indicators for Quality Management and Control of Tertiary Hospitals by National Health Commission |
| 67 | Incidence of Surgical Wound Dehiscence in Surgical Patients | Not defined in public document | Facility(Hospital) | No risk adjustment or risk stratification | Inpatient/Hospital | Outcome | Quality surveillance and improvement | Generic | Indicators for Quality Management and Control of Tertiary Hospitals by National Health Commission |
| 68 | Postoperative Sudden Death Incidence in Surgical Patients | Not defined in public document | Facility(Hospital) | No risk adjustment or risk stratification | Inpatient/Hospital | Outcome | Quality surveillance and improvement | Generic | Indicators for Quality Management and Control of Tertiary Hospitals by National Health Commission |
| 69 | Surgical Complications in Deceased Surgical Patients | Not defined in public document | Facility(Hospital) | No risk adjustment or risk stratification | Inpatient/Hospital | Outcome | Quality surveillance and improvement | Generic | Indicators for Quality Management and Control of Tertiary Hospitals by National Health Commission |
| 70 | Postoperative Respiratory Failure Incidence in Surgical Patients | Not defined in public document | Facility(Hospital) | No risk adjustment or risk stratification | Inpatient/Hospital | Outcome | Quality surveillance and improvement | Generic | Indicators for Quality Management and Control of Tertiary Hospitals by National Health Commission |
| 71 | Incidence of Postoperative Physiological/Metabolic Disorders in Surgical Patients | Not defined in public document | Facility(Hospital) | No risk adjustment or risk stratification | Inpatient/Hospital | Outcome | Quality surveillance and improvement | Generic | Indicators for Quality Management and Control of Tertiary Hospitals by National Health Commission |
| 72 | Incidence of Anesthesia-Related Complications in Surgical Patients | Not defined in public document | Facility(Hospital) | No risk adjustment or risk stratification | Inpatient/Hospital | Outcome | Quality surveillance and improvement | Generic | Indicators for Quality Management and Control of Tertiary Hospitals by National Health Commission |
| 73 | Incidence of Foreign Object Retention During Surgery | Not defined in public document | Facility(Hospital) | No risk adjustment or risk stratification | Inpatient/Hospital | Outcome | Quality surveillance and improvement | Generic | Indicators for Quality Management and Control of Tertiary Hospitals by National Health Commission |
| 74 | Cesarean Section Rate | Not defined in public document | Facility(Hospital) | No risk adjustment or risk stratification | Inpatient/Hospital | Outcome | Quality surveillance and improvement | Obstetric | Indicators for Quality Management and Control of Tertiary Hospitals by National Health Commission |
| 75 | Percentage of Antibacterial Prophylaxis in Clean Surgical Procedures | Not defined in public document | Facility(Hospital) | No risk adjustment or risk stratification | Inpatient/Hospital | Process | Quality surveillance and improvement | Generic | Indicators for Quality Management and Control of Tertiary Hospitals by National Health Commission |
| 76 | Average duration of antibiotic prophylaxis in Clean Surgical Procedures | Not defined in public document | Facility(Hospital) | No risk adjustment or risk stratification | Inpatient/Hospital | Process | Quality surveillance and improvement | Generic | Indicators for Quality Management and Control of Tertiary Hospitals by National Health Commission |
| 77 | Percentage of patients undergoing clean surgery receiving antibiotics within 0.5-2.0 hours before the surgery | Not defined in public document | Facility(Hospital) | No risk adjustment or risk stratification | Inpatient/Hospital | Process | Quality surveillance and improvement | Generic | Indicators for Quality Management and Control of Tertiary Hospitals by National Health Commission |
| 78 | Percentage of patients receiving antibiotics within 0.5-2.0 hours prior to hip joint replacement surgery | Not defined in public document | Facility(Hospital) | No risk adjustment or risk stratification | Inpatient/Hospital | Process | Quality surveillance and improvement | Orthopedic | Indicators for Quality Management and Control of Tertiary Hospitals by National Health Commission |
| 79 | Percentage of patients receiving antibiotics within 0.5-2.0 hours prior to knee joint surgery | Not defined in public document | Facility(Hospital) | No risk adjustment or risk stratification | Inpatient/Hospital | Process | Quality surveillance and improvement | Orthopedic | Indicators for Quality Management and Control of Tertiary Hospitals by National Health Commission |
| 80 | Percentage of patients receiving antibiotics within 0.5-2.0 hours prior to uterine fibroid resection | Not defined in public document | Facility(Hospital) | No risk adjustment or risk stratification | Inpatient/Hospital | Process | Quality surveillance and improvement | Cancer | Indicators for Quality Management and Control of Tertiary Hospitals by National Health Commission |
| 81 | Agreement rate between preoperative diagnosis and postoperative pathological diagnosis for malignant tumors | Not defined in public document | Facility(Hospital) | No risk adjustment or risk stratification | Inpatient/Hospital | Process | Quality surveillance and improvement | Cancer | Indicators for Quality Management and Control of Tertiary Hospitals by National Health Commission |
| 82 | Volume of Surgical Patients | Not defined in public document | Facility(Hospital) | No risk adjustment or risk stratification | Inpatient/Hospital | Structure | Quality surveillance and improvement | Generic | Indicators for Quality Management and Control of Tertiary Hospitals by National Health Commission |
| 83 | Antimicrobial Prophylaxis Therapy Rate for Class I Surgical Wounds | Not defined in public document | Facility(Hospital) | No risk adjustment or risk stratification | Inpatient/Hospital | Process | Quality surveillance and improvement | Generic | Monitoring Indicators of Surgical Services for Quality and Safety Improvement by National Health Commission |
| 84 | Intraoperative Active Warming Rate | Not defined in public document | Facility(Hospital) | No risk adjustment or risk stratification | Inpatient/Hospital | Process | Quality surveillance and improvement | Generic | Monitoring Indicators of Surgical Services for Quality and Safety Improvement by National Health Commission |
| 85 | Intraoperative Hypothermia Incidence Rate | Not defined in public document | Facility(Hospital) | No risk adjustment or risk stratification | Inpatient/Hospital | Process | Quality surveillance and improvement | Generic | Monitoring Indicators of Surgical Services for Quality and Safety Improvement by National Health Commission |
