# Supplementary material for: Performance Measurement for Surgery at National Level in China
Source: Health Care Sci. 2026 Apr 15;5(2):109–16. doi: 10.1002/hcs2.70066 (PMC13109843; doi:10.1002/hcs2.70066)
Supplement: Supplementary file 2 — Table S2: Performance Measures in the United States. [file HCS2-5-109-s003.docx]

| **Table S2 Performance Measures for Surgery Endorsed by Official Agency in the United States** | | | | | | | | | | | | |
| --- | --- | --- | --- | --- | --- | --- | --- | --- | --- | --- | --- | --- |
| No | Measure | Data Source | | Level of Analysis | Risk  Adjustment | Setting of Care | Domains of Donabedian conceptual model | | Current Use | Surgical Specialty | Steward | Status |
| 1 | Risk-Adjusted Postoperative Renal Failure | | Registry data | Clinician: Group/  Practice; Facility | Statistical risk model | Inpatient/  Hospital | Outcome | Public Reporting; Quality Improvement (Internal to the specific organization) | | Cardiac | The Society of Thoracic Surgeons | Endorsed |
| 2 | Risk-Adjusted Surgical Re-exploration | | Registry data | Clinician: Group/  Practice; Facility | Statistical risk model | Inpatient/  Hospital | Outcome | Public Reporting | | Cardiac | The Society of Thoracic Surgeons | Endorsed |
| 3 | Beta Blockade at Discharge | | Registry data | Clinician: Group/  Practice Facility | No risk adjustment or risk stratification | Inpatient/  Hospital | Process | Public Reporting Quality Improvement (Internal to the specific organization) Quality Improvement with Benchmarking (external benchmarking to multiple organizations) | | Cardiac | The Society of Thoracic Surgeons | endorsed |
| 4 | Anti-Lipid Treatment Discharge | | Registry data | Clinician: Group/Practice Facility | No risk adjustment or risk stratification | Inpatient/  Hospital | Process | Public Reporting | | Cardiac | The Society of Thoracic Surgeons | endorsed |
| 5 | Risk-Adjusted Operative Mortality for CABG | | Registry data | Clinician: Group/Practice; Facility | Statistical risk model | Inpatient/  Hospital | Outcome | Public Reporting; Quality Improvement (Internal to the specific organization) | | Cardiac | The Society of Thoracic Surgeons | endorsed |
| 6 | Risk-Adjusted Operative Mortality for Aortic Valve Replacement (AVR) | | Registry data | Clinician: Group/Practice; Facility | Statistical risk model | Inpatient/  Hospital | Outcome | Public Reporting | | Cardiac | The Society of Thoracic Surgeons | endorsed |
| 7 | Risk-Adjusted Operative Mortality for Mitral Valve (MV) Replacement | | Registry data | Clinician: Group/Practice; Facility | Statistical risk model | Inpatient/  Hospital | Outcome | Public Reporting | | Cardiac | The Society of Thoracic Surgeons | endorsed |
| 8 | Risk-Adjusted Operative Mortality for Mitral Valve (MV) Replacement + CABG Surgery | | Registry data | Clinician: Group/Practice; Facility | Statistical risk model | Inpatient/  Hospital | Outcome | Public Reporting | | Cardiac | The Society of Thoracic Surgeons | endorsed |
| 9 | Risk-Adjusted Operative Mortality for Aortic Valve Replacement (AVR) + CABG Surgery | | Registry data | Clinician: Group/Practice; Facility | Statistical risk model | Inpatient/  Hospital | Outcome | Public Reporting | | Cardiac | The Society of Thoracic Surgeons | endorsed |
| 10 | Preoperative Beta Blockade | | Registry data | Clinician: Group/Practice Facility | No risk adjustment or risk stratification | Inpatient/  Hospital | Process | Payment Program Public Reporting Quality Improvement (Internal to the specific organization) Quality Improvement with Benchmarking (external benchmarking to multiple organizations) | | Cardiac | The Society of Thoracic Surgeons | endorsed |
| 11 | Risk-Adjusted Postoperative Prolonged Intubation (Ventilation) | | Registry data | Clinician: Group/Practice; Facility | Statistical risk model | Inpatient/  Hospital | Outcome | Public Reporting; Quality Improvement (Internal to the specific organization) | | Cardiac | The Society of Thoracic Surgeons | endorsed |
| 12 | Risk-Adjusted Deep Sternal Wound Infection | | Registry data | Clinician: Group/Practice; Facility | Statistical risk model | Inpatient/  Hospital | Outcome | Public Reporting | | Cardiac | The Society of Thoracic Surgeons | endorsed |
| 13 | Risk-Adjusted Stroke/Cerebrovascular Accident | | Registry data | Clinician: Group/Practice; Facility | Statistical risk model | Inpatient/  Hospital | Outcome | Public Reporting; Quality Improvement (Internal to the specific organization) | | Cardiac | The Society of Thoracic Surgeons | endorsed |
| 14 | Use of Internal Mammary Artery (IMA) in Coronary Artery Bypass Graft (CABG) | | Registry data | Clinician: Group/Practice Facility | No risk adjustment or risk stratification | Inpatient/  Hospital | Process | Payment Program Public Reporting Quality Improvement (Internal to the specific organization) Quality Improvement with Benchmarking (external benchmarking to multiple organizations) | | Cardiac | The Society of Thoracic Surgeon | [Endorsed](https://p4qm.org/taxonomy/term/11) |
| 15 | Participation in a Systematic National Database for General Thoracic Surgery | | Registry data | Clinician: Group/Practice | No risk adjustment or risk stratification | Inpatient/  Hospital | Structure | Public Reporting Quality Improvement (Internal to the specific organization) Quality Improvement with Benchmarking (external benchmarking to multiple organizations) | | Thoracic | The Society of Thoracic Surgeons | endorsed |
| 16 | STS CABG Composite Score | | Registry data | Clinician: Group/Practice; Facility | Statistical risk model | Inpatient/  Hospital | Outcome | Public Reporting | | Cardiac | The Society of Thoracic Surgeons | endorsed |
| 17 | Surgical Volume for Pediatric and Congenital Heart Surgery: Total Programmatic Volume and Programmatic Volume Stratified by the 5 STAT Mortality Categories | | Registry data | Clinician: Group/Practice | Stratification by risk category/subgroup | Inpatient/  Hospital | Structure | Public Reporting | | Cardiac | The Society of Thoracic Surgeons | endorsed |
| 18 | Operative Mortality Stratified by the 5 STAT Mortality Categories | | Registry data | Clinician: Group/Practice | Stratification by risk category/subgroup | Inpatient/  Hospital | Outcome | Public Reporting | | Cardiac | The Society of Thoracic Surgeons | endorsed |
| 19 | Participation in a National Database for Pediatric and Congenital Heart Surgery | | Registry data | Clinician: Group/Practice Other | No risk adjustment or risk stratification | Inpatient/  Hospital | Structure | Public Reporting Quality Improvement (Internal to the specific organization) Quality Improvement with Benchmarking (external benchmarking to multiple organizations) | | Cardiac | The Society of Thoracic Surgeons | endorsed |
| 20 | Risk-Adjusted Operative Mortality for Mitral Valve (MV) Repair | | Registry data | Clinician: Group/Practice; Facility | Statistical risk model | Inpatient/  Hospital | Outcome | Public Reporting | | Cardiac | The Society of Thoracic Surgeons | endorsed |
| 21 | Risk-Adjusted Operative Mortality for Mitral Valve (MV) Repair + CABG Surgery | | Registry data | Clinician: Group/Practice; Facility | Statistical risk model | Inpatient/  Hospital | Outcome | Public Reporting | | Cardiac | The Society of Thoracic Surgeons | endorsed |
| 22 | Statin Therapy at Discharge after Lower Extremity Bypass (LEB) | | Registry data | Clinician: Group/Practice Clinician: Individual Facility | No risk adjustment or risk stratification | Inpatient/  Hospital | Process | Payment Program | | Vascular Surgery | Society for Vascular Surgery | endorsed |
| 23 | Rate of Open Repair of Abdominal Aortic Aneurysms (AAA) Where Patients Are Discharged Alive | | Registry data | Clinician: Group/Practice; Clinician: Individual; Facility | No risk adjustment or risk stratification | Inpatient/  Hospital | Outcome | Payment Program; Quality Improvement (Internal to the specific organization) | | [Vascular Surgery](https://p4qm.org/taxonomy/term/1536) | Society for Vascular Surgery | endorsed |
| 24 | Postoperative Stroke or Death in Asymptomatic Patients undergoing Carotid Endarterectomy | | Registry data | Clinician: Group/Practice; Clinician: Individual; Facility | No risk adjustment or risk stratification | Inpatient/  Hospital | Outcome | Payment Program | | Vascular Surgery | Society for Vascular Surgery | endorsed |
| 25 | Postoperative Stroke or Death in Asymptomatic Patients undergoing Carotid Artery Stenting (CAS) | | Registry data | Clinician: Individual; Facility | No risk adjustment or risk stratification | Inpatient/  Hospital | Outcome | Payment Program; Quality Improvement (Internal to the specific organization) | | Vascular Surgery | Society for Vascular Surgery | endorsed |
| 26 | Hospital-level risk-standardized complication rate (RSCR) following elective primary total hip arthroplasty (THA) and/or total knee arthroplasty (TKA) | | Claims data; Enrollment Data | [Facility](https://p4qm.org/taxonomy/term/881) | Statistical risk model | Inpatient/  Hospital | Outcome | Payment Program; Public Reporting | | Orthopedic | Centers for Medicare & Medicaid Services | endorsed |
| 27 | Hospital-level 30-day risk-standardized readmission rate (RSRR) following elective primary total hip arthroplasty (THA) and/or total knee arthroplasty (TKA) | | Claims data; Enrollment Data | [Facility](https://p4qm.org/taxonomy/term/881) | Statistical risk model | Inpatient/  Hospital | Outcome | Payment Program; Public Reporting | | Orthopedic | Centers for Medicare & Medicaid Services | endorsed |
| 28 | Performing cystoscopy at the time of hysterectomy for pelvic organ prolapse to detect lower urinary tract injury | | Paper Medical Records Registry data | Clinician: Group/Practice Clinician: Individual | No risk adjustment or risk stratification | Inpatient/  Hospital | Process | Quality Improvement (Internal to the specific organization) Quality Improvement with Benchmarking (external benchmarking to multiple organizations) | | Genitourinary | American Urogynecology Society | endorsed |
| 29 | Hospital 30-day, all-cause, risk-standardized mortality rate (RSMR) following coronary artery bypass graft (CABG) surgery | | Claims data | Facility | Statistical risk model | Inpatient/  Hospital | Outcome |  | | Cardiac | Centers for Medicare & Medicaid Services | endorsed |
| 30 | STS Aortic Valve Replacement (AVR) Composite Score | | Registry data | Clinician: Group/Practice; Facility | Statistical risk model | Inpatient/  Hospital | Outcome | Public Reporting; Quality Improvement (Internal to the specific organization) | | Cardiac | The Society of Thoracic Surgeons | endorsed |
| 31 | STS Aortic Valve Replacement (AVR) + Coronary Artery Bypass Graft (CABG) Composite Score | | Registry data | Clinician: Group/Practice; Facility | Statistical risk model | Inpatient/  Hospital | Outcome | Public Reporting; Quality Improvement (Internal to the specific organization); Quality Improvement with Benchmarking (external benchmarking to multiple organizations) | | Cardiac | The Society of Thoracic Surgeons | endorsed |
| 32 | Risk-Adjusted Operative Mortality for Pediatric and Congenital Heart Surgery | | Registry data | Clinician: Group/Practice | Statistical risk model | Inpatient/  Hospital | Outcome | Public Reporting | | Cardiac | The Society of Thoracic Surgeons | endorsed |
| 33 | Hospital Visits after Hospital Outpatient Surgery | | Claims data; Enrollment Data | Facility | Statistical risk model | Outpatient Services | Outcome | Payment Program; Public Reporting | | General | Centers for Medicare & Medicaid Services | endorsed |
| 34 | STS Individual Surgeon Composite Measure for Adult Cardiac Surgery | | Registry data | Clinician: Individual | Statistical risk model | Inpatient/  Hospital | Outcome | Quality Improvement (Internal to the specific organization); Quality Improvement with Benchmarking (external benchmarking to multiple organizations) | | Cardiac | The Society of Thoracic Surgeons | endorsed |
| 35 | STS Mitral Valve Repair/Replacement (MVRR) Composite Score | | Registry data | Clinician: Group/Practice; Facility | Statistical risk model | Inpatient/  Hospital | Outcome | Public Reporting; Quality Improvement (Internal to the specific organization); Quality Improvement with Benchmarking (external benchmarking to multiple organizations) | | Cardiac | The Society of Thoracic Surgeons | endorsed |
| 36 | STS Mitral Valve Repair/Replacement (MVRR) + Coronary Artery Bypass Graft (CABG) Composite Score | | Registry data | Clinician: Group/Practice; Facility | Statistical risk model | Inpatient/  Hospital | Outcome | Public Reporting; Quality Improvement (Internal to the specific organization); Quality Improvement with Benchmarking (external benchmarking to multiple organizations) | | Cardiac | The Society of Thoracic Surgeons | endorsed |
| 37 | Facility-Level 7-Day Hospital Visits after General Surgery Procedures Performed at Ambulatory Surgical Centers | | Claims data; Enrollment Data | Facility | Statistical risk model | Outpatient Services | Outcome |  | | General | Centers for Medicare & Medicaid Services | endorsed |
| 38 | Risk-standardized complication rate (RSCR) following elective primary total hip arthroplasty (THA) and/or total knee arthroplasty (TKA) for Merit-based Incentive Payment System (MIPS) Eligible Clinicians and Eligible Clinician Groups | | Claims data; Enrollment Data | Clinician: Group/Practice; Clinician: Individual | Statistical risk model | Inpatient/  Hospital; Outpatient Services | Outcome | Payment Program | | Orthopedic | Centers for Medicare & Medicaid Services | endorsed |
| 39 | Hospital 90-Day, All-Cause, Risk-Standardized Mortality Rate (RSMR) Following Coronary Artery Bypass Graft (CABG) Surgery | | Claims data | Facility | Statistical risk model | Inpatient/  Hospital | Outcome | Payment Program | | Cardiac | Centers for Medicare & Medicaid Services | endorsed |
| 40 | Clinician-Level and Clinician Group-Level Total Hip Arthroplasty and/or Total Knee Arthroplasty (THA and TKA) Patient-Reported Outcome-Based Performance Measure (PRO-PM) | | Claims data; Electronic Health Data; Instrument-Based Data; Other | Clinician: Group/Practice; Clinician: Individual | Statistical risk model | Inpatient/  Hospital | Outcome |  | | Orthopedic | Centers for Medicare & Medicaid Services | endorsed |
| 41 | Participation in a Systematic Database for Cardiac Surgery | | Registry data | Clinician: Group/Practice Facility Other | No risk adjustment or risk stratification | Inpatient/  Hospital | Structure | Public Reporting Quality Improvement (Internal to the specific organization) Quality Improvement with Benchmarking (external benchmarking to multiple organizations) | | Cardiac | The Society of Thoracic Surgeons | Endorsed with Reserve Status |
| 42 | Anti-Platelet Medication at Discharge | | Registry data | Clinician: Group/Practice Facility | No risk adjustment or risk stratification | Inpatient/  Hospital | Process |  | | Cardiac | The Society of Thoracic Surgeons | Endorsed with Reserve Status |
| 43 | Selection of Antibiotic Prophylaxis for Cardiac Surgery Patients | | Registry data | Clinician: Group/Practice Facility | No risk adjustment or risk stratification | Inpatient/  Hospital | Process | Quality Improvement (Internal to the specific organization) Quality Improvement with Benchmarking (external benchmarking to multiple organizations) | | Cardiac | The Society of Thoracic Surgeons | Endorsed with Reserve Status |
| 44 | Duration of Antibiotic Prophylaxis for Cardiac Surgery Patients | | Other Data Source Paper Patient Medical Records Registry data | Clinician: Group/Practice Clinician: Individual Facility | No risk adjustment or risk stratification | Inpatient/  Hospital | Process | Payment Program Quality Improvement (Internal to the specific organization) Quality Improvement with Benchmarking (external benchmarking to multiple organizations) | | Cardiac | The Society of Thoracic Surgeons | Endorsed with Reserve Status |
| 45 | Radiation therapy is administered within 1 year (365 days) of diagnosis for women under age 70 receiving breast conserving surgery for breast cancer | | Registry data | Facility | No risk adjustment or risk stratification | Inpatient/  Hospital | Process | Public Reporting Quality Improvement (Internal to the specific organization) Regulatory and Accreditation Programs | | Cancer | American College of Surgeons | Endorsed |
| 46 | Perioperative Care: Selection of Prophylactic Antibiotic: First- OR Second-Generation Cephalosporin | | Claims Data | Clinician: Group/Practice Clinician: Individual | No risk adjustment or risk stratification | Inpatient/  Hospital Outpatient Services | Process | Payment Program Public Reporting Quality Improvement (Internal to the specific organization) | | Generic | American Society of Plastic Surgeons | Endorsed with Reserve Status |
| 47 | Timing of Prophylactic Antibiotics - Administering Physician | | Claims Data Electronic Health Records: Electronic Health Records Other Data Source Paper Patient Medical Records Registry data | Clinician: Group/Practice Clinician: Individual Facility | No risk adjustment or risk stratification | Inpatient/  Hospital Outpatient Services | [Process](https://p4qm.org/taxonomy/term/96) | Public Reporting Quality Improvement (Internal to the specific organization) | | General | American Society of Anesthesiologists | Endorsed with Reserve Status |
| 48 | Perioperative Care: Discontinuation of Prophylactic Parenteral Antibiotics (Non-Cardiac Procedures) | | Claims Data | Clinician: Group/Practice Clinician: Individual | No risk adjustment or risk stratification | Inpatient/  Hospital Outpatient Services | Process | Payment Program Public Reporting Quality Improvement (Internal to the specific organization) | | Generic | Physician Consortium for Performance Improvement | Endorsed with Reserve Status |
| 49 | Perioperative Anti-platelet Therapy for Patients undergoing Carotid Endarterectomy | | Registry data | Clinician: Group/Practice Clinician: Individual Facility | No risk adjustment or risk stratification | Inpatient/  Hospital Outpatient Services | Process | Medicare Physician Quality Reporting System (PQRS) Physician Feedback/Quality and Resource Use Reports (QRUR) Physician Value-Based Payment Modifier (VBM) | | Vascular Surgery | Society for Vascular Surgery | Endorsed |
| 50 | PC-02 Cesarean Birth | | Electronic Health Records: Electronic Health Records Other Data Source Paper Patient Medical Records | Facility; Other | No risk adjustment or risk stratification | Inpatient/  Hospital | Outcome | Public Reporting Regulatory and Accreditation Programs | | Obstetric | The Joint Commission | Endorsed |
| 51 | ePC-02 Cesarean Birth | | Electronic Health Data; Electronic Health Records | Facility | No risk adjustment or risk stratification | Inpatient/  Hospital | Outcome | Quality Improvement (Internal to the specific organization); Quality Improvement with Benchmarking (external benchmarking to multiple organizations); Regulatory and Accreditation Programs | | Obstetric | The Joint Commission | Endorsed |
| 52 | Prophylactic Antibiotic Received Within One Hour Prior to Surgical Incision | | Claims Data Other Data Source Paper Patient Medical Records | Facility Other | No risk adjustment or risk stratification | Inpatient/  Hospital | Process | Payment Program Public Reporting Quality Improvement with Benchmarking (external benchmarking to multiple organizations) Regulatory and Accreditation Programs | | Generic | Centers for Medicare & Medicaid Services | Endorsed with Reserve Status |
| 53 | Prophylactic Antibiotic Selection for Surgical Patients | | Claims Data Other Data Source Paper Patient Medical Records | Facility Other | No risk adjustment or risk stratification | Inpatient/  Hospital | Process | Payment Program Public Reporting Quality Improvement with Benchmarking (external benchmarking to multiple organizations) Regulatory and Accreditation Programs | | Generic | Centers for Medicare & Medicaid Services | Endorsed with Reserve Status |
| 54 | Prophylactic Antibiotics Discontinued Within 24 Hours After Surgery End Time | | Claims Data Other Data Source Paper Patient Medical Records | Facility Other | No risk adjustment or risk stratification | Inpatient/  Hospital | Process | Payment Program Public Reporting Quality Improvement with Benchmarking (external benchmarking to multiple organizations) Regulatory and Accreditation Programs | | Generic | Centers for Medicare & Medicaid Services | Endorsed with Reserve Status |
| 55 | Cataracts: Complications within 30 Days Following Cataract Surgery Requiring Additional Surgical Procedures | | Registry data | Clinician: Group/Practice; Clinician: Individual | No risk adjustment or risk stratification | Inpatient/  Hospital; Outpatient Services | Outcome |  | | Ophthalmology | American Academy of Ophthalmology | Endorsed |
| 56 | Cataracts: Complications within 30 Days Following Cataract Surgery Requiring Additional Surgical Procedures | | Electronic Health Records: Electronic Health Records | Clinician: Group/Practice; Clinician: Individual | No risk adjustment or risk stratification | Inpatient/  Hospital; Outpatient Services | Outcome | End-Stage Renal Disease Quality Incentive Program (ESRD QIP) | | Ophthalmology | American Academy of Ophthalmology | Endorsed |
| 57 | Cataracts: 20/40 or Better Visual Acuity within 90 Days Following Cataract Surgery | | Registry data | Clinician: Group/Practice; Clinician: Individual | No risk adjustment or risk stratification | Inpatient/  Hospital; Outpatient Services | Outcome |  | | Ophthalmology | American Academy of Ophthalmology | Endorsed |
| 58 | Cataracts: 20/40 or Better Visual Acuity within 90 Days Following Cataract Surgery | | Electronic Health Records: Electronic Health Records | Clinician: Group/Practice; Clinician: Individual | No risk adjustment or risk stratification | Inpatient/  Hospital; Outpatient Services | Outcome |  | | Ophthalmology | American Academy of Ophthalmology | Endorsed |
| 59 | American College of Surgeons – Centers for Disease Control and Prevention (ACS-CDC) Harmonized Procedure Specific Surgical Site Infection (SSI) Outcome Measure | | Electronic Health Data; Electronic Health Records; Other Data Source; Paper Patient Medical Records | Facility; Other ; Population: Regional and State | Other | Inpatient/  Hospital | Outcome | Payment Program; Public Health/Disease Surveillance; Public Reporting; Regulatory and Accreditation Programs | | Generic | Centers for Disease Control and Prevention | Endorsed |
| 60 | Risk-Adjusted Coronary Artery Bypass Graft (CABG) Readmission Rate | | Claims Data; Registry data | Facility | Statistical risk model | Inpatient/  Hospital | Outcome | Payment Program; Public Reporting; Quality Improvement (Internal to the specific organization) | | Cardiac | The Society of Thoracic Surgeons | Endorsed |
| 61 | Hospital 30-day, all-cause, unplanned, risk-standardized readmission rate (RSRR) following coronary artery bypass graft (CABG) surgery | | Claims Data; Enrollment Data | Facility | Statistical risk model | Inpatient/  Hospital | Outcome | Public Reporting | | Cardiac | Centers for Medicare & Medicaid Services | Endorsed |
| 62 | Informed, Patient Centered (IPC) Hip and Knee Replacement Surgery | | Instrument-Based Data | Clinician: Group/Practice | No risk adjustment or risk stratification | Outpatient Services | Outcome | Professional Certification or Recognition Program; Quality Improvement (Internal to the specific organization) | | Orthopedic | Massachusetts General Hospital | Endorsed |
| 63 | STS Lobectomy for Lung Cancer Composite Score | | Other Data Source; Registry data | Facility | Statistical risk model | Inpatient/  Hospital | Outcome | Public Reporting; Quality Improvement (Internal to the specific organization); Quality Improvement with Benchmarking (external benchmarking to multiple organizations) | | Thoracic | The Society of Thoracic Surgeons | Endorsed |
| 64 | Facility-Level 7-Day Hospital Visits after Urology Procedures Performed at Ambulatory Surgical Centers | | Claims data; Enrollment Data | Facility | Statistical risk model |  | Outcome | Public Reporting | | Genitourinary | Centers for Medicare & Medicaid Services | Endorsed |
| 65 | Hospital Visits after Orthopedic Ambulatory Surgical Center Procedures | | Claims Data | Facility | Statistical risk model | Outpatient Services | Outcome | Public Reporting | | Orthopedic | Centers for Medicare & Medicaid Services | Endorsed |
| 66 | Hospital-level, risk-standardized payment associated with a 90-day episode of care for elective primary total hip and/or total knee arthroplasty (THA/TKA) | | Claims data; Enrollment Data | Facility | Statistical risk model | Inpatient/  Hospital | Outcome |  | | Orthopedic | Centers for Medicare & Medicaid Services | Endorsed |
| 67 | Knee Arthroplasty | | Claims Data; Enrollment Data; Other Data Source | Clinician: Group/Practice; Clinician: Individual | Stratification by risk category/subgroup |  | Outcome | Payment Program | | Orthopedic | Centers for Medicare & Medicaid Services | Endorsed |
| 68 | Discouraging the routine use of occupational and/or supervised physical therapy after carpal tunnel release | | Claims Data | Clinician: Individual Facility | No risk adjustment or risk stratification | Inpatient/  Hospital Outpatient Services | [Process](https://p4qm.org/taxonomy/term/96) | Payment Program Quality Improvement (Internal to the specific organization) Quality Improvement with Benchmarking (external benchmarking to multiple organizations) | | Orthopedic | American Academy of Orthopaedic Surgeons | Endorsed |
| 69 | 30 Day All-cause Risk Standardized Mortality Odds Ratio following Transcatheter Aortic Valve Replacement (TAVR) | | Registry data | Facility | Statistical risk model | Inpatient/  Hospital | Outcome | Public Reporting; Quality Improvement with Benchmarking (external benchmarking to multiple organizations); Regulatory and Accreditation Programs | | Cardiac | American College of Cardiology | Endorsed |
| 70 | Hospital-Level, Risk-Standardized Patient-Reported Outcomes Following Elective Primary Total Hip and/or Total Knee Arthroplasty (THA/TKA) | | Claims Data; Instrument-Based Data | Facility | Statistical risk model | Inpatient/  Hospital | Outcome | Payment Program; Quality Improvement (Internal to the specific organization) | | Orthopedic | Centers for Medicare & Medicaid Services | Endorsed |
| 71 | 30-day Risk Standardized Morbidity and Mortality Composite following Transcatheter Aortic Valve Replacement (TAVR) | | Registry data | Facility | Statistical risk model | Inpatient/  Hospital | Outcome | Public Reporting; Quality Improvement with Benchmarking (external benchmarking to multiple organizations) | | Cardiac | American College of Cardiology | Endorsed |
| 72 | Ventriculoperitoneal (VP) shunt malfunction rate in children | | Claims Data | Clinician: Group/Practice; Clinician: Individual | Statistical risk model | Urgent Care - Ambulatory Ambulatory Care: Clinician Office Inpatient/Hospital Other Care Setting (Please specify) | Outcome | Payment Program | | Orthopedic | Centers for Medicare & Medicaid Services | Endorsed |
| 73 | Non-Emergent Coronary Artery Bypass Graft (CABG) Measure | | Claims Data | Clinician: Group/Practice; Clinician: Individual | Stratification by risk category/subgroup | Inpatient/  Hospital | Outcome | Payment Program | | Cardiac | Centers for Medicare & Medicaid Services | Endorsed |
| 74 | Lumbar Spine Fusion for Degenerative Disease, 1-3 Levels Measure | | Claims Data | Clinician: Group/Practice; Clinician: Individual | Stratification by risk category/subgroup | Urgent Care - Ambulatory Inpatient/  Hospital Other Care Setting (Please specify) | Outcome | Payment Program | | Orthopedic | Centers for Medicare & Medicaid Services | Endorsed |
