# Supplementary material for: Performance Measurement for Surgery at National Level in China
Source: Health Care Sci. 2026 Apr 15;5(2):109–16. doi: 10.1002/hcs2.70066 (PMC13109843; doi:10.1002/hcs2.70066)
Supplement: Supplementary file 3 — Table Caption for S1 and S2. [file HCS2-5-109-s002.docx]

**Captions**

Table S1: Performance Measures in China. 85 measures were included in this analysis. Characteristics of 85 included surgical performance measures from Chinese national quality monitoring databases. Measures were aggregated from two sources: the Quality Management and Control Indicators for Tertiary Hospitals (n=77) and the Monitoring Indicators of Surgical Services for Quality and Safety Improvement (n=8).

Table S2: Performance Measures in the United States. A total of 156 surgery-related performance measures received NQF endorsement. Currently, 85 have lost endorsement status, while 74 retain valid endorsement—64 as active and 10 as reserve. The present review encompassed exclusively measures with valid endorsement (active or reserve).
